# Validation of Pediatric Emergency Care Applied Research Network (PECARN) rule in children with minor head trauma

**DOI:** 10.1371/journal.pone.0262102

**Published:** 2022-01-18

**Authors:** Sooje Cho, Soyun Hwang, Jae Yun Jung, Young Ho Kwak, Do Kyun Kim, Jin Hee Lee, Jin Hee Jung, Joong Wan Park, Hyuksool Kwon, Dongbum Suh

**Affiliations:** 1 Department of Emergency Medicine, Seoul National University Hospital, Seoul, Korea; 2 Department of Emergency Medicine, Seoul National University Bundang Hospital, Seongnam, Korea; 3 Department of Emergency Medicine, Seoul Metropolitan Government Seoul National University Boramae Medical Center, Seoul, Korea; Kaohsuing Medical University Hospital, TAIWAN

## Abstract

The Pediatric Emergency Care Applied Research Network (PECARN) rule is commonly used for predicting the need for computed tomography (CT) scans in children with mild head trauma. The objective of this study was to validate the PECARN rule in Korean children presenting to the pediatric emergency department (PED) with head trauma. This study was a multicenter, retrospective, observational cohort study in two teaching PEDs in Korea between August 2015 and August 2016. In this observational study, 448 patients who visited PEDs were included in the final analysis. Risk stratification was performed with clinical decision support software based on the PECARN rule, and decisions to perform CT scans were subsequently made. Patients were followed-up by phone call between 7 days and 90 days after discharge from the PED. The sensitivity and specificity were analyzed. The sensitivity was 100% for all age groups, and no cases of clinically important traumatic brain injury (ciTBI) were identified in the very-low-risk group. CT scans were performed for 14.7% of patients in this study and for 33.8% in the original PECARN study. The PECARN rule successfully identified low-risk patients, and no cases of ciTBI were missed despite the reduced proportion of patients undergoing CT scans.

## Introduction

In the USA, more than 600,000 children visit the emergency department annually with head trauma, which is a leading cause of death [1]. The incidence of head trauma varies by country, with a range between 47 and 280 per 100,000 children [2]. However, less than 10% of patients with head trauma have traumatic brain injury, and less than 1% need neurosurgical interventions [3, 4]. CT is the modality of choice for diagnosing traumatic brain injury, but ionizing radiation from CT scans can lead to malignancies, and the risk is higher in children [5–7].

Due to the high prevalence of childhood head injury but low risk of serious outcomes in the absence of traumatic brain injury, studies have focused on identifying children at low risk for traumatic brain injury and therefore reducing the performance of unnecessary CT scans. Such studies were the basis for the development of clinical decision rules (CDRs), including the Canadian Assessment of Tomography for Childhood Head Injury (CATCH) [8], Children’s Head Injury Algorithm for the Prediction of Important Clinical Events (CHALICE) [9], and Pediatric Emergency Care Applied Research Network (PECARN) [1]. The PECARN rule is the most commonly used CDR due to its higher sensitivity and specificity.

The PECARN rule has been validated in several countries both retrospectively and prospectively, [10–12] and some studies have shown a reduced CT scan rate after the implementation of the PECARN rule in clinical practice[13–15]. However, it has not yet been properly validated in Korean children. The aim of this study was to validate the PECARN rule in Korean pediatric patients who visited the pediatric emergency department (PED) presenting with head trauma.

## Methods

### Study design

This study was a retrospective cohort study performed in PEDs in two teaching hospitals in Korea. Approximately 20,000 patients visit these two PEDs annually. All patients were evaluated and treated by a resident emergency physician paired with an attending pediatric emergency specialist.

The two hospitals adopted the PECARN rule in August 2015 as a part of a quality assurance campaign between August 2015 and August 2016, which focused on reducing CT scans, especially in pediatric patients, but also on minimizing the rate of missed cases of clinically important traumatic brain injury (ciTBI). Clinical decision support software that was specifically developed for the campaign was used to calculate the risk of ciTBI based on the PECARN rule by entering the patient’s age, symptoms, and signs. Whenever a child younger than 19 years of age visits the PED with head trauma, the attending doctors utilize the software and rely on the PECARN algorithm to determine whether to order CT scans.

If the child’s risk of ciTBI was high by the PECARN algorithm, CT scan was performed, and if the risk of ciTBI was intermediate, whether or not to perform CT scan was basically based on the physician’s judgment, but sufficient information was provided to the parents and final decision about CT scan was made after discussion. In the case of low risk of ciTBI, the physicians explained to the parents that ciTBI risk was low and CT was not recommended. However, in the case of intermediate risk and low risk, there is a possibility of simple skull fracture or minor brain hemorrhage which cannot be ruled out by the PECARN rule, but it was sufficiently explained to the parents that the child can be observed at home but should return to the PED in case of worsening symptoms.

When discharging low risk patients without CT scans, patients and guardians were educated regarding possible symptoms and signs associated with traumatic brain injury and were instructed to visit PED when needed. An animation video explaining when to revisit PED and possible symptoms and signs associated with traumatic brain injury was developed and sent to all patients’ guardians via mobile message. Between 7 days and 90 days after discharge, to ensure no missing TBI, a follow-up phone call was made by a designated nurse and asked whether the patient was diagnosed with traumatic brain injury after being discharged from the ED.

This study was approved by the Seoul National University Hospital Institutional Review Board and Seoul National University Bundang Hospital Institutional Review Board. The need to obtain informed consent was waived because this study was retrospective and involved no risk to the patients, as the PECARN rule has been validated by the CDR and widely adopted.

### Participants

To retrospectively validate the PECARN rule, we included patients younger than 19 years of age who presented with head trauma within 24 hours of the injury to two PEDs between August 2015 and August 2016. Eligible patients were extracted from the registry of an Emergency Department-based Injury In-depth Surveillance System, which is a nationwide database of injured patients visiting EDs that was established in 2006 by the Korea Centers for Disease Control and Prevention.

The exclusion criteria and other definitions were the same as those in the original PECARN study [1]. Patients with a Glasgow Coma Scale (GCS) score less than 14, previous history of neurological disease, or trivial injury mechanism (ground level fall, collision with a stationary object, no signs or symptoms of head trauma other than scalp abrasions and lacerations) were excluded. ciTBI was defined as death from traumatic brain injury, neurosurgical intervention for traumatic brain injury, intubation of more than 24 h for traumatic brain injury and hospital admission of 2 nights or more for traumatic brain injury in association with evidence of traumatic brain injury on CT.

### Data collection

The following variables were collected and analyzed. Information about patient characteristics, including age, sex, ED visit time, mechanism of injury, formal report of brain CT scan, final diagnosis, and disposition after ED visit, was collected. In addition, findings in the review of systems and on physical examination related to the PECARN rule (agitation, somnolence, repetitive questioning, slow response to verbal communication, palpable skull fracture, scalp hematoma and its location, history of loss of consciousness and its duration, signs of basal skull fracture, and abnormal behavior reported by parents) were coded.

### Outcome

The collected data were analyzed, and the patients were stratified into groups according to risk, as previously defined by the PECARN group. Patients were divided into two groups by age, since the original PECARN rule has different criteria for two age groups, namely, patients less than 2 years old and patients 2 years old or older. The primary outcome was the sensitivity and specificity of the original PECARN rule for identifying patients at low risk of ciTBI in both age groups.

### Statistical analysis

Continuous variables are presented as medians with interquartile ranges, and categorical variables are presented as numbers and frequencies. To compare variables between participant groups, a chi-square test was performed for categorical variables, and a t-test was performed for continuous variables. The sensitivity, specificity, and positive and negative predictive values (PPV and NPV) for the diagnostic performance of the PECARN rule were calculated. A receiver operating curve was generated, and the area under the curve (AUC) was calculated. A P value less than 0.05 was considered statistically significant. All statistical analyses were performed using SPSS for Windows, version 23.0 (IBM Corp.).

## Results

**Fig 1:**
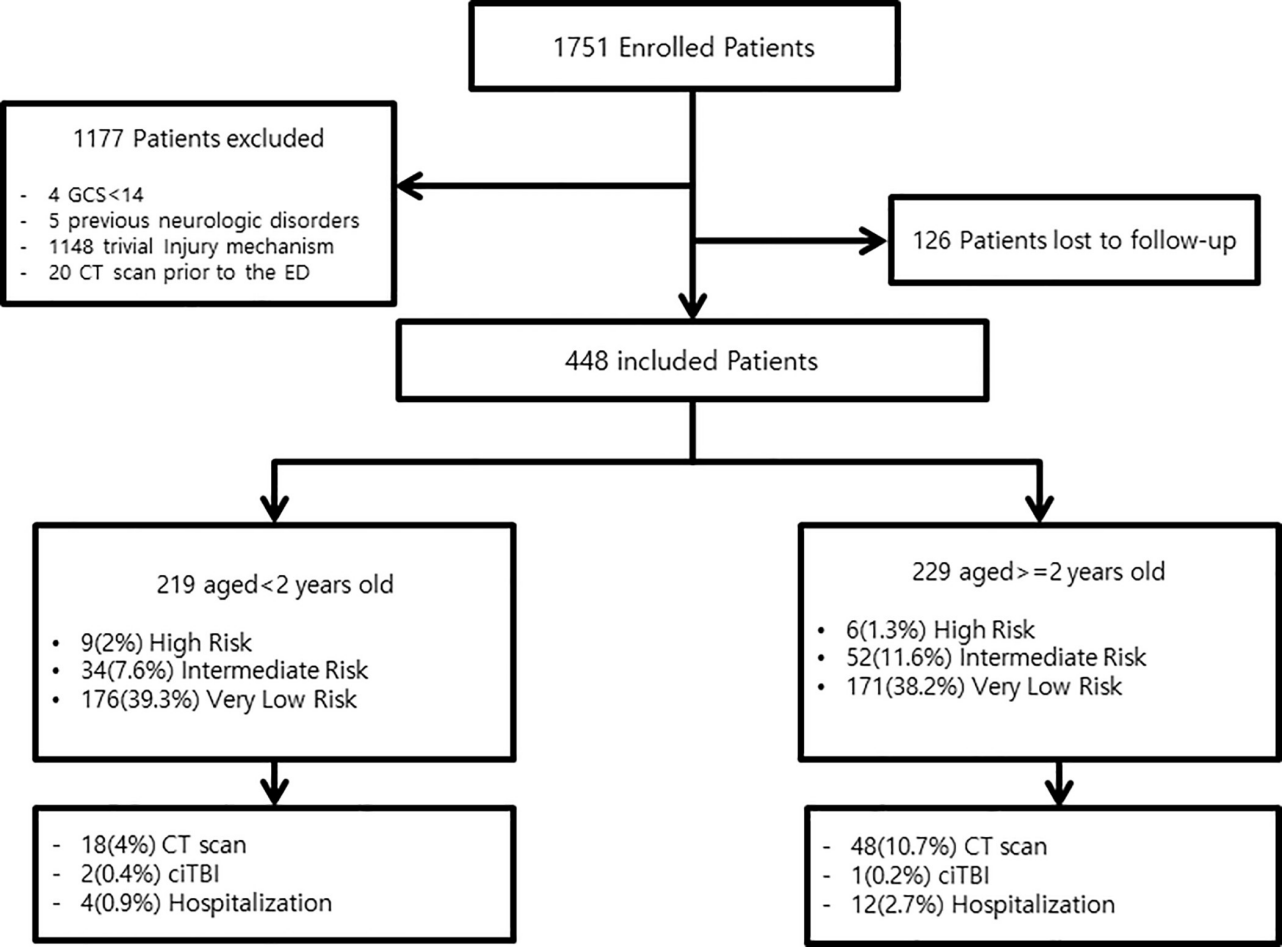
Flowchart of the study.

A total of 1751 patients visited two PEDs with head trauma during the study period. A total of 1148 patients were excluded because of trivial injury mechanisms, 4 patients were excluded because they were evaluated as having GCS scores less than 14, 5 patients were excluded because of previous neurologic disorders, and 20 patients were excluded because they had visited another hospital before visiting the participating hospital. In total, 126 patients were lost to follow-up. The flowchart of the patients is shown in Fig 1.

Of the 448 patients, 219 (48.9%) were younger than 2 years old, and 260 (58%) were male. The clinical characteristics of the patients compared with those in the PECARN original validation study are presented in Table 1. CT scans were performed for 66 (14.7%) patients, while they were performed for 33.8% of the patients in the original PECARN study. ciTBI was found in 3 patients. Two patients had epidural hematoma (EDH) and associated skull fracture, and one had skull fracture without intracranial hemorrhage. All three patients were admitted to the general ward for two nights or more for close observation and were discharged without any complications. No patients underwent neurosurgery. Among the patients who were discharged after assessment in the PED without CT scans, none were later diagnosed with ciTBI according to the follow-up telephone calls.

**Table 1:** Clinical characteristics of patients compared with PECARN original validation study.

Characteristics	Study cohort, n = 448 n(%)	PECARN, n = 8627 n(%)
Age(IQR^a^), y^b^	2.7(0–4)	7.1*
Male	260 (58)	NR
<2 years of age	219 (48.9)	2216 (25.7)
Risk of ciTBI		
*High risk*	15 (3.3)	1468 (17)
Glasgow Coma Scale = 14	2 (0.4)	255 (3)
Altered Mental Status	9 (2.0)	1082 (12.6)
Signs of basilar skull fracture	1 (0.2)	51 (0.8)
Palpable skull fracture	3 (0.7)	80 (3.6)
*Intermediate risk*	86 (19.2)	2183 (25.3)
Severe mechanism of injury	10 (2.2)	1271 (14.9)
Non frontal hematoma	25 (5.6)	361 (16.5)
Loss of consciousness ≥ 5 seconds	8 (1.8)	1160 (14.1)
Vomiting	33 (7.4)	1050 (12.3)
Severe headache	4 (0.9)	146 (2.8)
Not acting normally	6 (1.3)	273 (12.7)
*Very low risk*	347 (77.4)	4976 (57.7)
CT	66 (14.7)	2917 (33.8)
Any positive findings on CT	39 (8.7)	184 (6.3)
ciTBI	3 (0.7)	88 (1)
Neurosurgery	0 (0)	16 (0.2)

^a^IQR interquartile range

^b^y years

*Mean age in derivation and validation cohort

**Fig 2:**
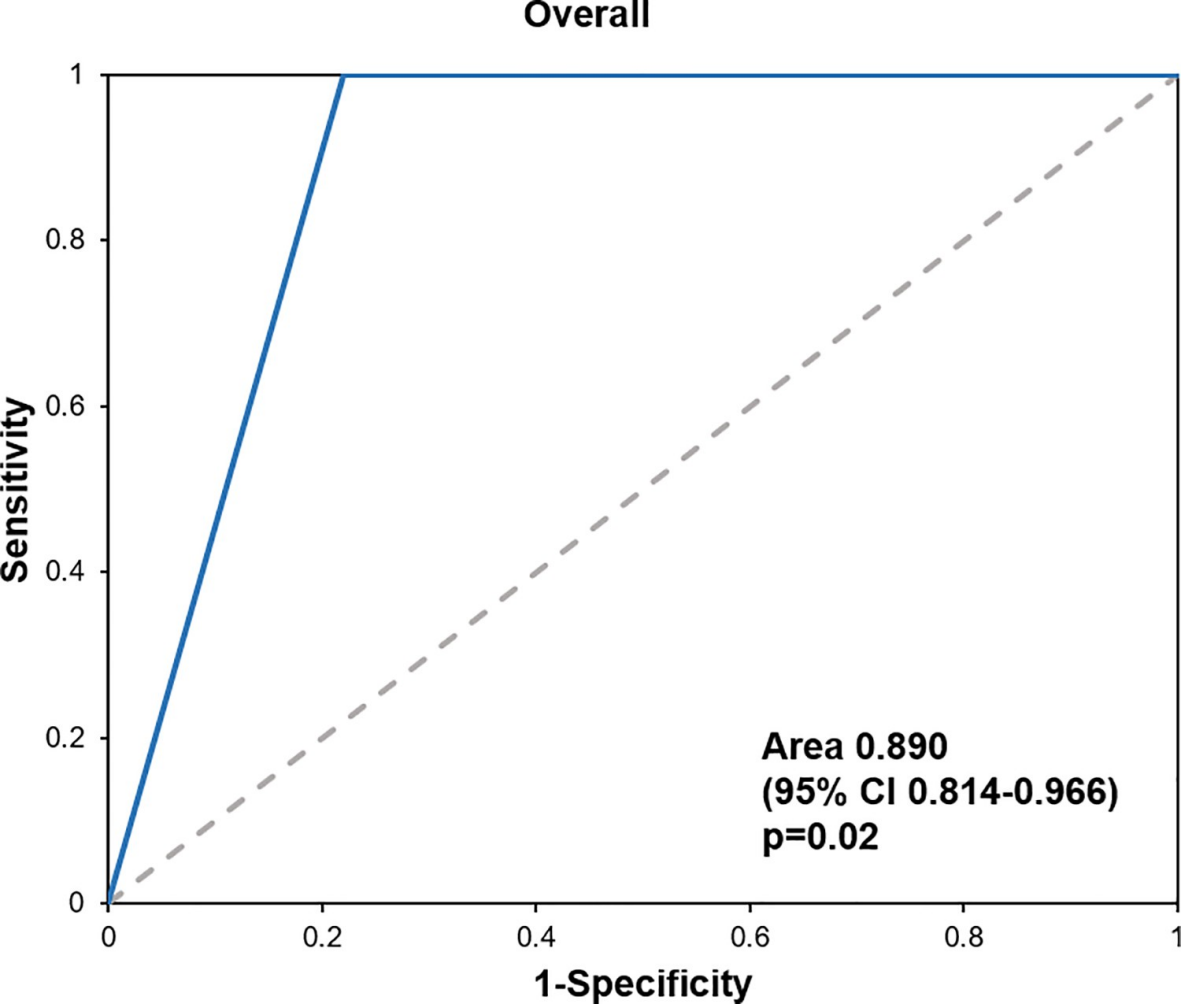
The ROC curves for the overall age group.

**Fig 3:**
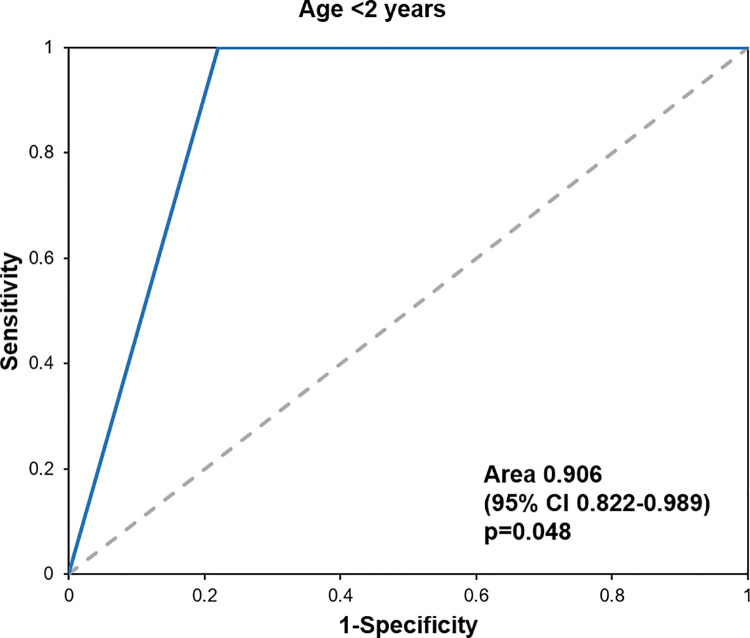
The ROC curves for patients younger than 2 years old.

**Fig 4:**
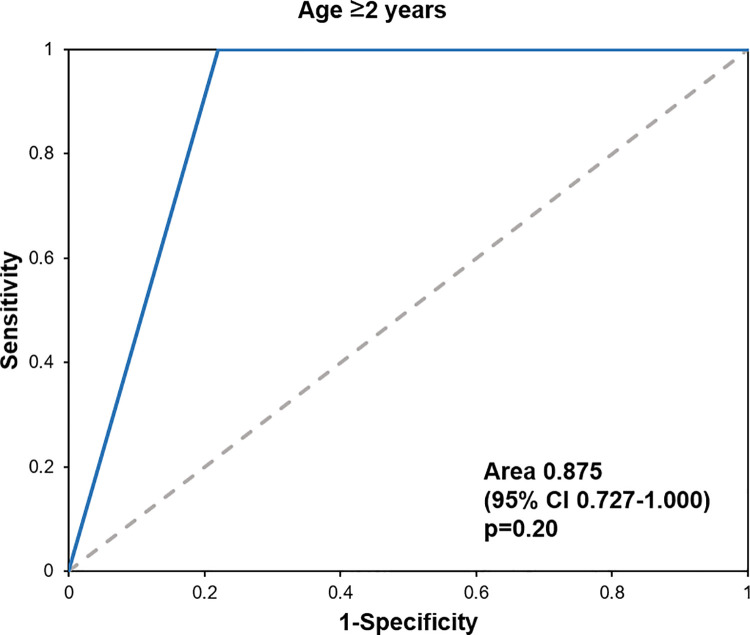
The ROC curves for patients aged 2 years of older.

The diagnostic accuracy of the PECARN rule was evaluated using the sensitivity, specificity, NPV, and PPV. The sensitivity was 100% for both age groups. There were no cases of ciTBI identified in the very-low-risk group. The data are presented in Table 2. The ROC curves for the overall age group are presented in Fig 2, those for patients younger than 2 years old are shown in Fig 3, and those for patients aged 2 years or older are presented in Fig 4.

**Table 2:** Diagnostic accuracy of the PECARN rule.

PECARN ciTBI risk group	ciTBI		Sensitivity (95% CI^a^)	Specificity (95% CI)	Negative predictive value (95% CI)	Positive predictive value (95% CI)
	Yes	No				
<2 years			100% (19.8%-100%)	81.1% (75.1%-86.0%)	100% (97.3%-100%)	4.7% (0.8%-17.1%)
Intermediate or high risk	2	41				
Very low risk	0	176				
≥2 years			100% (5.1%-100%)	74.6% (68.4%-80.1%)	100% (97.2%-100%)	1.7% (0.1%-10.5%)
Intermediate or high risk	1	57				
Very low risk	0	171				
Overall			100% (31.0%-100%)	78.0% (73.8%-81.7%)	100% (98.6%-100%)	3.0% (0.8%-9.1%)
Intermediate or high risk	3	98				
Very low risk	0	347				

^a^CI confidence interval

## Discussion

In this study, the PECARN rule was adopted in two PEDs to reduce the rate of CT scans in pediatric patients presenting with minor head trauma. The PECARN rule was an accurate and safe method of identifying patients at low risk for ciTBI. The sensitivity and NPV of the PECARN rule were 100% in both age groups. Therefore, the PECARN rule was validated as a safe CDR in our study population.

In this study, CT scans were performed in 14.7% of the patients, which was lower than the 33.8% of the patients who underwent CT scans in the original PECARN study. A nationwide study [16] in a Korean population showed that 17.5% of a pediatric population underwent CT scans, which was similar to our data. The lower proportion of patients undergoing CT scans may be due to the lower risk of ciTBI in our population. As EDs are easily accessible and national health insurance covers more than 99% of population [17], patients with less severe injury might have visited ED more often. Additionally, there is a possibility of physicians being biased towards not ordering CT scans because the PECARN rule is a well-known clinical decision rule that has already been internally and externally [18] validated and had been adopted into routine clinical practice in the two hospitals as part of the quality assurance campaign to reduce CT scans. However, despite the lower proportion of patients who underwent CT scans, there were no missed cases of ciTBI. This supports of possibility of safely further reducing the performance of CT scans.

Several studies have attempted to validate the PECARN rule in the Korean population [19–21]. However, they all had limitations. Some studies were performed with patients who had already undergone CT scans, and they had limitations with regard to evaluating the safety of the PECARN rule because they did not include patients assessed as having a low risk of ciTBI who were discharged without having undergone CT scans. Another study that analyzed the effects of introducing the PECARN rule into clinical practice in one hospital [21] used the original PECARN study design. The patients were classified into groups based on the PECARN rule, and the sensitivity and specificity were evaluated. However, there was a possibility of missed cases of TBI because the majority of patients were lost to follow-up.

In contrast, in this study, most patients who visited the PED with head trauma responded to the telephone follow-up, enabling the outcome to be verified. Therefore, we believe that our study is a relatively more effective evaluation of the PECARN rule than the previous studies.

This study has several limitations. First, this study was performed in only two hospitals, so there was the possibility of selection bias. However, although the two hospitals are tertiary hospitals, referrals are not needed to visit such hospitals in Korea. Therefore, it can be assumed that our study population was not markedly different from the general population.

Although the sensitivity was 100% in both age groups, our data showed wide range 95% confidence intervals, which were 19.8%-100% in patients younger than 2 years of age and 5.1%-100% in patients aged 2 years or older. This was due to the small numbers of patients with ciTBI in the intermediate- and high-risk groups. As mentioned before, our study population had an overall lower risk of ciTBI than the population in the original PECARN study; thus, we failed to recruit a sufficient number of patients to detect significance. Given the low incidence of ciTBI in our population, a larger, multicenter study is needed.

Additionally, we followed up the patients via telephone 7~90 days after discharge from the PED, but 126 patients were lost to follow-up. However, one recent study [22] showed that missed intracranial injuries are rare when the PECARN rule is used. A total of 1.5% of the patients returned to the PED, only 0.19% underwent neuroimaging, and no patients needed neurosurgery or intensive care. There might have been missed ciTBI cases among the patients who were lost to follow-up in our study, but the possibility is extremely low.

As this was a registry-based retrospective study, the medical records that were originally written by attending PED physicians were later reviewed by researchers; therefore, signs and symptoms could have been misinterpreted [23].

Finally, this study lacked an evaluation of physically abused children. These two hospitals have special teams for abused children, and attending physicians are obligated to alert the team via the electronic medical system if abuse is suspected as a mechanism of injury. There were no reports of abused children in our study population. Therefore, we could not analyze the diagnostic accuracy of the PECARN rule in abused children. The clinical report from the American Academy of Pediatrics [24] suggested that all infants and children with head trauma suspected of having been caused by abuse should undergo head CT or MRI. Additionally, the author of the original PECARN study suggested that the rule should not be applied to cases of abusive head trauma [25], and in a study validating the PECARN rule in a Japanese population, the PECARN rule had a sensitivity of 100% for identifying patients younger than 2 years of age who were at low risk for ciTBI after sustaining minor head trauma without physical abuse. However, in an analysis including patients with head trauma caused by abuse, the sensitivity was only 85.7% [10]. Therefore, when applying the PECARN rule to decide whether to perform CT scans in pediatric patients with head trauma, careful consideration must be given to a possible abusive mechanism of injury.

## Conclusion

The PECARN rule is a safe and reliable CDR that can be used to identify children at low risk for ciTBI in South Korea. There is a possibility that the proportion of patients undergoing CT scans could be further reduced because there were no missed cases of ciTBI despite the lower proportion of patients undergoing CT scans than reported in the original PECARN study.

## Supporting information

S1 Data Complete dataset of current study. (XLSX)
